# Differences in breast cancer-risk factors between screen-detected and non-screen-detected cases (MCC-Spain study)

**DOI:** 10.1007/s10552-021-01511-4

**Published:** 2021-11-24

**Authors:** Marta Hernández-García, Ana Molina-Barceló, Mercedes Vanaclocha-Espi, Óscar Zurriaga, Beatriz Pérez-Gómez, Nuria Aragonés, Pilar Amiano, Jone M. Altzibar, Gemma Castaño-Vinyals, María Sala, María Ederra, Vicente Martín, Inés Gómez-Acebo, Carmen Vidal, Adonina Tardón, Rafael Marcos-Gragera, Marina Pollán, Manolis Kogevinas, Dolores Salas

**Affiliations:** 1 Cancer and Public Health Area, Foundation for the Promotion of Health and Biomedical Research of Valencia Region(FISABIO), Avda. Catalunya 21, 46020 Valencia, Spain; 2 Preventive Medicine and Public Health, Food Sciences, Toxicology and Forensic Medicine Department, University of Valencia, Avda. Vicent Andrés Estellés, s/n, 46100 Burjassot, Valencia Spain; 3 Joint Research Unit on Rare Diseases, FISABIO-UVEG, Avda. Catalunya 21, 46020 Valencia, Spain; 4 Directorate General of Public Health, Avda. Catalunya 21, 46020 Valencia, Spain; 5 Spanish Consortium for Research on Epidemiology and Public Health (CIBERESP), Instituto de Salud Carlos III, Madrid, Spain; 6 Department of Epidemiology of Chronic Diseases, National Center of Epidemiology, Carlos III Health Institute (ISCIII), Avda. Monforte de Lemos 5, 28029 Madrid, Spain; 7 Epidemiology Section, Public Health Division, Department of Health of Madrid, C/San Martín de Porres, 6, 28035 Madrid, Spain; 8 Epidemiology of Chronic and Communicable Diseases Group, Biodonostia Health Research Institute, Doctor Begiristain, s/n, 20014 San Sebastián, Spain; 9 Sub Directorate for Public Health and Addictions of Gipuzkoa, Ministry of Health of the Basque Government, 2013, San Sebastian, Spain; 10 ISGlobal, Barcelona Institute for Global Health, Doctor Aiguader 88, 08003 Barcelona, Spain; 11 IMIM (Hospital del Mar Medical Research Institute), Doctor Aiguader 88, 08003 Barcelona, Spain; 12 Universitat Pompeu Fabra (UPF), Plaça de la Mercè, 10-12, 08002 Barcelona, Spain; 13 Research Network on Health Services in Chronic Diseases (REDISSEC), Barcelona, Spain; 14 Navarra Public Health Institute, C/ Leyre, 15, 31003 Navarra, Spain; 15 IdiSNA, Navarra Institute for Health Research, C/Irunlarrea, 3, 31008 Pamplona, Spain; 16 The Research Group in Gene - Environment and Health Interactions (GIIGAS), Biomedicine Institute (IBIOMED), University of León, Vegazana Campus, s/n, 24071 León, Spain; 17 Cantabria University– IDIVAL, C/Cardenal Herrera Oria, s/n, Santander, 39011 Cantabria, Spain; 18 Cancer Screening Unit, Catalan Institute of Oncology, Duran I Reynals Hospital, Avda. de La Gran Via de L′Hospitalet, 199-203, L′Hospitalet de Llobregat, 08908 Barcelona, Spain; 19 Early Detection of Cancer Research Group, EPIBELL Program, Bellvitge Biomedical Research Institute, Avda. de La Granvia de L′Hospitalet, 199, L′Hospitalet de Llobregat, 08908 Barcelona, Spain; 20 Oncology Institute (IUOPA), University of Oviedo, Edificio Santiago Gascón, Campus El Cristo B, 33006 Oviedo, Spain; 21 Epidemiology Unit and Girona Cancer Registry, Oncology Coordination Plan, Department of Health, Autonomous Government of Catalonia, Catalan Institute of Oncology. Sant Ponç, Avda de França, 0, 17007 Girona, Spain; 22 Descriptive Epidemiology, Genetics and Cancer Prevention Group, [Girona Biomedical Research Institute] IDIBGI, C/ del Dr. Castany, s/n, Salt, 17190 Girona, Spain; 23 National Center of Epidemiology Directorate, Carlos III Health Institute (ISCIII), Avda. Monforte de Lemos 5, 28029 Madrid, Spain

**Keywords:** Breast neoplasm, Risk factors, Early detection of cancer, Phenotype

## Abstract

**Purpose:**

The variation in breast cancer (BC)-risk factor associations between screen-detected (SD) and non-screen-detected (NSD) tumors has been poorly studied, despite the interest of this aspect in risk assessment and prevention. This study analyzes the differences in breast cancer-risk factor associations according to detection method and tumor phenotype in Spanish women aged between 50 and 69.

**Methods:**

We examined 900 BC cases and 896 controls aged between 50 and 69, recruited in the multicase–control MCC-Spain study. With regard to the cases, 460 were detected by screening mammography, whereas 144 were diagnosed by other means. By tumor phenotype, 591 were HR+, 153 were HER2+, and 58 were TN. Lifestyle, reproductive factors, family history of BC, and tumor characteristics were analyzed. Logistic regression models were used to compare cases vs. controls and SD vs. NSD cases. Multinomial regression models (controls used as a reference) were adjusted for case analysis according to phenotype and detection method.

**Results:**

TN was associated with a lower risk of SD BC (OR 0.30 IC 0.10–0.89), as were intermediate (OR 0.18 IC 0.07–0.44) and advanced stages at diagnosis (OR 0.11 IC 0.03–0.34). Nulliparity in postmenopausal women and age at menopause were related to an increased risk of SD BC (OR 1.60 IC 1.08–2.36; OR 1.48 IC 1.09–2.00, respectively). Nulliparity in postmenopausal women was associated with a higher risk of HR+ (OR 1.66 IC 1.15–2.40). Age at menopause was related to a greater risk of HR+ (OR 1.60 IC 1.22–2.11) and HER2+ (OR 1.59 IC 1.03–2.45) tumors.

**Conclusion:**

Reproductive risk factors are associated with SD BC, as are HR+ tumors. Differences in BC-risk factor associations according to detection method may be related to prevailing phenotypes among categories.

**Supplementary Information:**

The online version contains supplementary material available at 10.1007/s10552-021-01511-4.

## Introduction

Breast cancer (BC) is the most common tumor among women worldwide. An estimated 2,261,419 new cases occurred globally in 2020 [1]. The main associated risk factors include nulliparity, early menarche, and late menopause (all three factors related to endogenous hormone exposure), as well as alcohol consumption, being overweight, obesity, and lack of physical activity [2–6]. High-penetrance gene mutations (BRCA1, BRCA2, TP53, PTEN) considerably increase BC risk, however, they only account for a small proportion of the disease burden given their low frequency in the population [2, 4, 5].

Breast cancer is a heterogeneous disease; it includes several entities with a different natural history and response to treatment. Breast tumors can be classified into phenotypes based on the expression of hormone receptors and human epidermal growth factor receptor 2 in tumor cells [7]. Reproductive behavior influences the development of HR+ (hormone receptor positive) tumors as it modifies estrogen levels, whereas lifestyle-related factors (body mass index [BMI], tobacco, alcohol, and physical activity) similarly affect different tumor subtypes [8–10]. In addition, HR+ breast carcinomas often have smaller tumors and are in an early stage at diagnosis, features that are associated with a better prognosis [9, 11]. In contrast, HER2+ (human epidermal growth factor receptor 2 positive) and TN (triple negative) carcinomas have larger tumors and are in a more advanced stage at diagnosis [9]. Additionally, TN is associated with aggressive metastatic breast cancer, partly due to its contribution to the rapid progression of the disease [12].

Through European Council Recommendation of 2 December 2003 on cancer screening [13], the EU advises the implementation of systematic population-based breast cancer screening programs as part of prevention strategies. By performing diagnostic tests on an asymptomatic population, screening allows for early detection of the disease, thereby helping to reduce the impact on mortality [14–16]. In Spain, these programs have been included in the Spanish National Health System’s service portfolio since 2014, although they have been progressively implemented since the 1990s in different regions. They consist of biennial mammography and the target population is women aged 50–69. According to 2017 data [17], coverage is approximately 85% and the participation rate is over 70%. Nevertheless, uptake is unequal among individuals; women living in the least deprived areas are more likely to participate in breast cancer screening programs than women living in the most deprived areas. This is not only due to their limited knowledge about mammography or misconceptions, but also to practical barriers such as difficulties attending mammography appointments or family commitments [18]. Apart from population-based screening programs, part of the target population participates in opportunistic breast cancer screening practices [19, 20]. These are individual, non-systematic population-based interventions carried out at the patient’s request or arising from a visit for another medical reason.

Tumor characteristics according to detection method have been previously studied. By comparison, non-screen-detected BC (either due to a failure to participate in screening programs or the appearance of interval cancer) is associated with more advanced stages at diagnosis, aggressive tumors, and poorer prognoses, whereas screen-detected tumors are more frequently diagnosed at an early stage, are of a small size, and express hormone receptors [21–24].

A previous study in the United States has shown differences in breast cancer-risk factor associations by the method of detection [25]. However, there are few studies on variation in breast cancer-risk factor associations between screen-detected and non-screen-detected tumors, and therefore there is little information available on this matter. This study examines whether the screening method affects risk factor associations, regardless of tumor phenotype.

Thus, we aim to analyze the differences in breast cancer-risk factor associations according to method of detection and tumor phenotype in a population of Spanish women aged 50–69.

## Material and methods

### Study population

MCC-Spain is a population-based multicase–control study aimed at investigating the influence of environmental and genetic factors on different tumors (gastric, colorectal, breast, prostate, and chronic lymphocytic leukemia) [26]. Cases and population controls were recruited between 2008 and 2013 in twelve Spanish regions. Incident cases were actively recruited at 23 collaborating hospitals and population controls were contacted by telephone and invited to take part in the study, after being randomly selected from primary care center lists within the catchment area of these 23 hospitals. Cases had no prior history of cancer and they were identified as soon as possible after the diagnosis was made through an active search that included regular visits to the collaborating hospital departments. Only histologically confirmed incident cancer cases, including breast cancer, were included in the study (International Classification of Diseases 10th Revision: C50, D05.1, D05.7).

Controls were frequency-matched to cases by age, sex, and region, ensuring that there was at least one control of the same sex and within the same five-year age interval for each case in each region.

Participants had resided within the hospital catchment areas for at least 6 months. Cases and controls answered an epidemiological questionnaire in a personal interview. The questionnaire (available at http://www.mccspain.org) focused on socio-demographic characteristics, lifestyle, medical history, and family history of cancer. Additionally, subjects were provided with a semi-quantitative Food Frequency Questionnaire (FFQ), which was a modified version of a previously validated instrument in Spain [27]. It estimated regular dietary intake during the previous year. Clinical and pathological information on the diagnosis and treatment of tumors was collected from hospital records.

We included 1.796 women in the study in this analysis, 896 controls and 900 BC cases. Inclusion criteria for cases were BC tumor histologically diagnosed during the recruitment period, home address in one of the collaborating hospitals’ catching area, age range 50–69. Cases exclusion criteria were BC diagnosis previous to patient recruitment period, inability to communicate in Spanish, and physical incapacity to take part in the study. By phenotype, 591 were HR+ tumors, 153 were HER2+, and 58 were TN (phenotype data were missing for 98 cases). By self-referred detection method, 460 cases were screen-detected either by organized or spontaneous screening mammography (average age 59,04 years old), and 144 cases were non-screen-detected (average age 57,88 years old). Detection method data were missing and/or inconsistent for 296 cases. The age range was defined in accordance with European Council recommendations on cancer screening programs [13].

### Definition of variables.

Detection method: BC cases were classified into screen-detected and non-screen-detected tumors, based on the answers provided in the epidemiological questionnaire. BC cases were considered screen-detected whenever participants declared that the tumor had been diagnosed by screening (organized or spontaneous screening mammography performed in the absence of symptoms) and, additionally, that this mammogram had been performed in the last 6 months. On the contrary, BC cases were classified as non-screen-detected when patients declared that the tumor had not been diagnosed by mammogram screening and, additionally, that they had not undergone a screening mammography in the last 6 months.

Using a simplified version of the St Gallen International Consensus [28], breast tumors were classified into the following phenotypes: HR+ (hormone receptor positive), HER2+ (human epidermal growth factor receptor 2 positive, regardless of hormone receptors), and TN (triple negative, i.e., negative for progesterone and estrogen receptors, and human epidermal growth factor receptor 2 negative). Other tumor characteristics included the stage at diagnosis (early − 0, IA, IB −; intermediate − IIA, IIB −; and advanced − IIIA, IIIB, IIIC, IV −), extension (in situ, invasive), and histology (ductal, lobular, other).

Epidemiological factors included reproductive factors (age at menarche, nulliparity, age at first birth, menopausal status, and age at menopause), history of breast cancer in first-degree relatives, and lifestyles factors (alcohol consumption over the year prior to the interview, tobacco use a month before the interview, leisure-time physical activity over a ten-year period up to two years prior to the interview, BMI at recruitment, and fruit and vegetable consumption over the year prior to the interview).

### Statistical analysis

A descriptive analysis of tumor characteristics and epidemiological factors was conducted for screen-detected/non-screen-detected cases, total cases/controls, screen-detected cases/controls, non-screen-detected cases/controls, HR+ cases/controls, HER2+ cases/controls, and TN/controls. Frequencies and percentages were calculated, and significant differences were tested using the Chi-square test for categorical variables and the Wilcoxon test for continuous variables.

Adjusted odds ratios (OR) and 95% confidence intervals (CI) were calculated using multivariate logistic regression models to analyze the association between epidemiological factors and overall BC risk, as well as to investigate the differences in screen-detected/non-screen-detected cases related to tumor characteristics and epidemiological factors.

A multinomial logistic regression model was conducted to examine whether risk factor associations vary according to method of detection. An additional multinomial logistic regression model was used to analyze variation in risk factor associations by tumor phenotype. In both models, controls were used as a reference group. The exponential multinomial logarithmic coefficient provided an estimate of relative risk, expressed here as the relative risk (RR) coefficient.

*p* values were considered statistically significant below 0.05. All statistical analyses were performed using the statistical software R (version 3.6.0). Analyses were adjusted by region, educational level, age, and menopausal status.

In order to assess the age-related risk factors of menopause and nulliparity, analyses were performed for the overall sample, for postmenopausal women, and for parous women.

## Results

### Sample description

Table 1 shows the results of the descriptive analysis. Statistically significant differences are observed in the association with epidemiological factors and tumor characteristics (only for cases), with heterogeneous results depending on the method of detection and phenotype.

**Table 1:** Sample description

				Cases by detection method		Cases by tumor phenotype		
	Controls		Cases	SD	NSD	HR+	HER2+	TN
	*n* (%)		*n* (%)	*n* (%)	*n* (%)	*n*(%)	*n*(%)	*n*(%)
Total	896		900	460	144	591	153	58
Tobacco use								
Never	491 (54.9)		509 (56.7)	258 (56.2)	88 (61.5)	331 (56.2)	91 (59.5)	36 (62.1)
Former smoker	227 (25.4)		244 (27.2)	127 (27.7)	33 (23.1)	157 (26.7)	41 (26.8)	18 (31)
Smoker	177 (19.8)		145 (16.1)	74 (16.1)	22 (15.4)	101 (17.1)	21 (13.7)	4 (6.9)
Alcohol, g/day (median, Q1–Q3)	1.9 (0.0–8.8)		2.0 (0.0–9.6)	1.9 (0.0–9.1)	1.8 (0.0–9.7)	2.4 (0.0–9.6)	1.2 (0.0–8.9)	1.2 (0.0–8.2)
BMI, kg/m^2^ (median, Q1–Q3)	25.2 (22.5–28.6)		26.2 (23.4–29.7)a	26.1 (23.4–29.2)b	26.7 (23.9–29.9)b	26.2 (23.5–29.7)c	26.0 (23.3–29.3)c	26.1 (23.3–29.8)
Physical activity (MET*h/week)								
Inactivity	346 (38.8)		346 (38.4)	146 (31.7)b	69 (47.9)b	225 (38.1)	68 (44.4)	18 (31.0)
Low activity	151 (16.9)		152 (16.9)	71 (15.4)	33 (22.9)	96 (16.2)	29 (19.0)	9 (15.5)
Moderate activity	134 (15.0)		114 (12.7)	55 (12.0)	16 (11.1)	75 (12.7)	19 (12.4)	8 (13.8)
High activity	261 (29.3)		288 (32.0)	188 (40.9)	26 (18.1)	195 (33)	37 (24.2)	23 (39.7)
Fruits and vegetables intake, g/day (median, Q1-Q3)	556.7 (393.7–718.4)		556.7 (393.6–718.4)	534.2 (368.6–742.9)	547.3 (387.8–732.5)	537.4 (364.2–756.3)	548.2 (406.9–729.8)	559.8 (456.3–769.6)
Age at menarche								
≤ 12 years	390 (45.3)		419 (47.0)	211 (46.1)	74 (52.1)	278 (47.4)	68 (45.0)	24 (41.4)
> 12 years	471 (54.7)		472 (53.0)	247 (53.9)	68 (47.9)	308 (52.6)	83 (55.0)	34 (58.6)
Nulliparity								
Yes	133 (14.9)		157 (17.5)	89 (19.3)b	20 (14.1)	486 (82.4)	125 (82.2)	49 (84.5)
No	762 (85.1)		741 (82.5)	371 (80.7)	122 (85.9)	104 (17.6)	27 (17.8)	9 (15.5)
Age at first birth^1^ (median, Q1-Q3)	25 (23–28)		25 (23–28)	26 (23–29)b	26 (22–27)b, d	25 (23–28)	25 (22–29)	26 (23–30)
Age at menopause^2^								
≤ 50 years	446 (65.3)		402 (57.3)a	208 (57.3)b	67 (62.6)	256 (56)c	71 (55.9)	32 (71.1)
> 50 years	237 (34.7)		299 (42.7)	155 (42.7)	40 (37.4)	201 (44)	56 (44.1)	13 (28.9)
Education level								
Less than primary school	113 (12.6)		126 (14.0)	59 (12.8)	23 (16.0)	91 (15.4)	20 (13.1)	6 (10.3)
Primary school	324 (36.2)		344 (38.2)	173 (37.6)	53 (36.8)	225 (38.1)	58 (37.9)	24 (41.4)
Secondary school	293 (32.7)		286 (31.8)	155 (33.7)	47 (32.6)	186 (31.5)	49 (32.0)	19 (32.8)
University and higher	166 (18.5)		144 (16.0)	73 (15.9)	21 (14.6)	89 (15.1)	26 (17.0)	9 (15.5)
Family history of BC								
Yes		89 (10.3)	118 (13.5)a	63 (14.2)b	12 (8.7)	499 (87.4)	128 (84.8)	49 (87.5)
No		778 (89.7)	754 (86.5)	381 (85.8)	126 (91.3)	72 (12.6)	23 (15.2)	7 (12.5)
Phenotype		–				–	–	–
HR+			591 (73.7)	303 (75.2)	85 (63.9)d			
HER2+			153 (19.1)	73 (18.1)	31 (23.3)			
TN			58 (7.2)	27 (6.7)	17 (12.8)			
Stage at diagnose		–						
Early			390 (54.2)	226 (61.6)	43 (37.7)d	273 (53.6)	53 (48.2)	20 (44.4)
Intermediate			245 (34.0)	109 (29.7)	49 (43.0)	175 (34.4)	41 (37.3)	17 (37.8)
Advanced			85 (11.8)	32 (8.7)	22 (19.3)	61 (12.0)	16 (14.5)	8 (17.8)
Extension		–						
In situ			111 (12.7)	64 (14.1)	11 (8.2)d	26 (4.5)	18 (12.0)	1 (1.8)e
Invasive			765 (87.3)	390 (85.9)	123 (91.8)	550 (95.5)	132 (88.0)	54 (98.2)
Histology		–						
Ductal			671 (86.3)	340 (86.3)	113 (87.6)	471 (84.1)	127 (96.2)	49 (87.5)e
Lobular			55 (7.1)	25 (6.3)	9 (7.0)	47 (8.4)	5 (3.8)	2 (3.6)
Other			52 (6.7)	29 (7.4)	7 (5.4)	42 (7.5)	0 (0.0)	5 (8.9)

^1^Only parous women

^2^Only postmenopausal women

^a^*p* value < 0.05, Chi-squared/ Wilcoxon test between control population and cases

^b^*p* value < 0.05, Chi-squared/ Wilcoxon test between control population and cancer subtype according to detection method

^c^*p* value < 0.05, Chi-squared/ Wilcoxon test between control population and cancer subtype according to phenotype

^d^*p* value < 0.05, Chi-squared/Wilcoxon test between cancer subtypes according to detection method (screen-detected vs. non-screen-detected)

^e^*p* value < 0,05, Chi-squared/Wilcoxon test between cancer subtypes according to phenotype (HR+, HER2+, and TN)

### Comparative analysis of tumor characteristics for screen-detected and non-screen-detected breast cancer

The multivariate logistic regression analysis for screen-detected/non-screen-detected tumors (Table 2) shows significant differences in terms of tumor characteristics. Screen-detected breast cancers are less likely to be diagnosed at intermediate (OR = 0.23; 95%CI 0.11–0.47) and advanced stages (OR = 0.14; 95%CI 0.06–0.36) than non-screen-detected breast cancers. Similar results are obtained for postmenopausal women and parous women, both for intermediate (OR = 0.18; 95%CI 0.07–0.44 and OR = 0.21; 95%CI 0.09–0.52, respectively) and advanced stages (OR = 0.11; 95%CI 0.03–0.34 and OR = 0.18; 95%CI 0.06–0.53, respectively). In comparison with HR+ tumors, TN tumors (OR = 0.30; 95%CI 0.10–0.89) are less likely to be screen-detected. Similar results are found for parous women (OR = 0.18; 95%CI 0.05–0.67).

**Table 2:** Tumor characteristics according to detection method, for the overall sample, postmenopausal women, and parous women

	Screen-detected BC vs. Non-screen-detected		
	Overall sample	Postmenopausal	Parous women
	OR^1^ (95%CI)	OR^2^ (95%CI)	OR^3^ (95%CI)
Phenotype (ref: HR+)			
HER2+	1.14 (0.47–2.77)	0.70 (0.25–1.93)	1.11 (0.38–3.24)
TN	0.30 (0.10–0.89)*	0.37 (0.10–1.34)	0.18 (0.05–0.67)*
Stage (ref: early)			
Intermediate	0.23 (0.11–0.47)*	0.18 (0.07–0.44)*	0.21 (0.09–0.52)*
Advanced	0.14 (0.06–0.36)*	0.11 (0.03–0.34)*	0.18 (0.06–0.53)*
Histology (ref: ductal)			
Lobular	1.26 (0.31–5.11)	2.85 (0.36–22.89)	3.11 (0.47–20.73)
Other	1.02 (0.25–4.21)	0.48 (0.09–2.52)	1.58 (0.23–10.70)

^1^OR adjusted for region, educational level, age, and epidemiological factors

^2^OR adjusted for region, educational level, age, epidemiological factors (except menopausal status)

^3^OR adjusted for region, educational level, age, epidemiological factors (except nulliparity)

*p value < 0.05

### Breast cancer risk by detection method

Table 3 shows breast cancer-risk factor associations according to detection method. The multinomial logistic regression model for the overall sample reveals that BMI was associated with a 5% greater risk of screen-detected BC (RR = 1.05; 95%CI 1.02–1.08) and a 4% greater risk of non-screen-detected BC (RR = 1.04; 95%CI 1.00–1.08). Similar results are shown for BMI among postmenopausal women and parous women, although in both cases we found a borderline significant trend for risk association with non-screen-detected breast cancers. Likewise, BMI is associated with a slightly higher risk of BC for overall breast cancer cases regardless of the detection method, as shown in the supplementary table.

**Table 3:** Breast cancer-risk variation by detection method. Multinomial logistic regression models (controls: reference groups); overall sample, postmenopausal women, and parous women

	Overall sample, RR^1^		Postmenopausal women RR^2^		Parous women RR^1^	
	SD	NSD	SD	NSD	SD	NSD
Tobacco use (ref: never)						
Ever	0.93 (0.70–1.22)	0.78 (0.50–1.22)	0.91 (0.67–1.24)	0.87 (0.51–1.49)	0.91 (0.67–1.23)	0.73 (0.44–1.20)
Alcohol consumption (g/day)	1.01 (0.99–1.02)	1.00 (0.99–1.02)	1.01 (0.99–1.02)	1.00 (0.98–1.02)	1.01 (0.99–1.02)	0.99 (0.97–1.02)
BMI	1.05 (1.02–1.08)*	1.04 (1.00–1.08)*	1.05 (1.02–1.08)*	1.05 (1.00–1.10)	1.04 (1.01–1.08)*	1.03 (0.99–1.07)
Physical Activity (ref: active)						
Inactive	0.87 (0.66–1.15)	1.36 (0.88–2.08)	0.92 (0.67–1.27)	1.29 (0.78–2.12)	0.90 (0.66–1.22)	1.34 (0.84–2.15)
Fruits and vegetables intake (g/day)	1.00 (0.99–1.00)	1.00 (0.99–1.00)	1.00 (0.99–1.00)	1.00 (0.99–1.00)	1.00 (0.99–1.00)	1.00 (0.99–1.00)
Age at menarche (ref: > 12 years)						
≤ 12 years	1.06 (0.82–1.37)	1.15 (0.75–1.75)	1.02 (0.76–1.37)	1.27 (0.78–2.07)	1.04 (0.78–1.39)	0.98 (0.62–1.55)
Nulliparity (ref: No)						
Yes	1.32 (0.94–1.86)	1.10 (0.61–1.98)	1.60 (1.08–2.36)*	1.28 (0.63–2.60)		
Age at menopause (ref: ≤ 50 years)						
> 50 years			1.48 (1.09–2.00)*	1.13 (0.68–1.85)		
Age at first birth					1.03 (0.99–1.07)	0.99 (0.95–1.03)
Family history of BC (ref: no)						
Yes	1.33 (0.90–1.96)	0.69 (0.31–1.51)	1.03 (0.65–1.64)	0.59 (0.24–1.50)	1.44 (0.94–2.20)	0.74 (0.31–1.72)

*p value < 0.05

^1^RR adjusted for region, educational level, and menopausal status; ^2^RR adjusted for region

Concerning reproductive factors, the association of nulliparity and age at menopause with breast cancer risk varied by detection method for postmenopausal women only, with a 60% (RR = 1.60; 1.08–2.36) and 48% (RR = 1.48; 95%CI 1.09–2.00) greater risk of screen-detected BC, respectively. Similar results for nulliparity and age at menopause are found in postmenopausal women for overall breast cancer cases, irrespective of the detection method (see the supplementary table).

### Breast cancer-risk variation by phenotype

Concerning lifestyle factors, the risk factor association analysis by phenotype (Table 4) reveals that BMI is related to a marginally increased risk of HR+ tumors for the overall sample (RR = 1.04; 95%CI 1.01–1.07), but also for postmenopausal women (RR = 1.05; 95%CI 1.02–1.08) and parous women (RR = 1.04; 95%CI 1.01–1.07). In the overall sample, we also found a probable increased risk of HER2+ (RR = 1.03; 95%CI 1.00–1.07) and TN (RR = 1.05; 95%CI 1.00–1.10) for BMI, with borderline significant results. Additionally, BMI is associated with a risk of TN (RR = 1.05; 95%CI 1.01–1.10) among postmenopausal women. All in all, the results indicated that BMI is related to a small increase in breast cancer risk, regardless of tumor phenotype.

**Table 4:** Breast cancer-risk variation by phenotype. Multinomial logistic regression models (controls: reference groups); overall sample, postmenopausal women, and parous women

	Overall sample, RR^1^			Postmenopausal women RR^2^			Parous women RR^1^		
	HR+	HER2+	TN	HR+	HER2+	TN	HR+	HER2+	TN
Tobacco use (ref: never)									
Ever	0.91 (0.71–1.17)	0.77 (0.51–1.17)	0.77 (0.41–1.42)	1.01 (0.76–1.34)	0.68 (0.42–1.09)	0.65 (0.32–1.32)	0.87 (0.66–1.15)	0.67 (0.42–1.07)	0.77 (0.39–1.54)
Alcohol cons. (g/day)	1.01 (1.00–1.02)*	1.00 (0.99–1.02)	1.00 (0.98–1.03)	1.01 (0.99–1.02)	1.00 (0.99–1.02)	0.99 (0.97–1.03)	1.01 (0.99–1.02)	1.01 (0.99–1.02)	1.00 (0.97–1.03)
BMI	1.04 (1.01–1.07)*	1.03 (1.00–1.07)*	1.05 (1.00–1.10)*	1.05 (1.02–1.08)*	1.02 (0.99–1.06)	1.05 (1.01–1.10)*	1.04 (1.01–1.07)*	1.03 (0.99–1.07)	1.04 (0.99–1.10)
Physical Act. (ref: active)									
Inactive	1.16 (0.91–1.49)	1.33 (0.90–1.98)	0.78 (0.41–1.49)	1.13 (0.85–1.50)	1.54 (0.98–2.41)	0.72 (0.34–1.53)	1.18 (0.90–1.55)	1.49 (0.96–2.31)	0.78 (0.38–1.61)
Fru. Veg. intake (g/day)	1.00 (1.00–1.00)	1.00 (0.99–1.00)	1.00 (0.99–1.00)	1.00 (1.00–1.00)	1.00 (0.99–1.00)	1.00 (0.99–1.00)	1.00 (0.99–1.00)	1.00 (0.99–1.00)	1.00 (0.99–1.00)
Age menarche (ref: > 12)									
≤ 12 years	1.05 (0.83–1.33)	1.02 (0.69–1.50)	1.10 (0.61–2.01)	1.07 (0.82–1.40)	1.04 (0.67–1.61)	1.02 (0.52–2.02)	1.04 (0.80–1.35)	1.09 (0.71–1.68)	1.17 (0.61–2.27)
Nulliparity (ref: No)									
Yes	1.30 (0.94–1.78)	1.16 (0.68–1.95)	1.23 (0.54–2.77)	1.66 (1.15–2.40)*	1.39 (0.76–2.55)	1.47 (0.60–3.61)			
Menopause (ref: ≤ 50)									
> 50 years				1.60 (1.22–2.11)*	1.59 (1.03–2.45)*	0.74 (0.36–1.54)			
Age at first birth							1.01 (0.98–1.04)	1.03 (0.98–1.07)	1.07 (1.01–1.12)*
Family history BC (ref: no)									
Yes	1.18 (0.82–1.69)	1.44 (0.83–2.51)	1.11 (0.45–2.73)	1.01 (0.66–1.55)	1.28 (0.67–2.42)	0.86 (0.29–2.56)	1.29 (0.87–1.92)	1.23 (0.65–2.33)	1.34 (0.53–3.39)

*p value < 0.05

^1^RR adjusted for region, educational level, and menopausal status

^2^RR adjusted for region

Table 4 also shows an increased risk, albeit very marginal, of HR+ tumors associated with alcohol consumption in the overall sample. In a similar manner, a borderline significant trend is observed for all phenotypes, also in postmenopausal and parous women.

A possible risk association between lack of physical activity and HER2+ breast cancer is observed in postmenopausal (RR = 1.54; 95%CI 0.98–2.41) and parous women (RR = 1.49; 95%CI 0.96–2.31), with borderline significant results.

Regarding reproductive factors, in postmenopausal women the strength of association of nulliparity varied according to phenotype, with an approximately 60% greater risk of HR+ BC (RR = 1.66; 95%CI 1.15–2.40). Age at menopause also changed risk associations depending on phenotype, with an approximately 60% greater risk of HR+ BC (RR = 1.60; 95%CI 1.22–2.11) and HER2+ BC (RR = 1.59; 95%CI 1.03–2.45). Additionally, in parous women, we also found that age at first birth was positively correlated to TN BC (RR = 1.07; 95%CI 1.01–1.12).

## Discussion

This study analyzes variation in breast cancer-risk factors according to method of detection and tumor phenotype. The results showed differences between the association of screen-detected and non-screen-detected breast cancer with the reproductive risk factors of age at menopause and nulliparity. In postmenopausal women, these factors were associated with a risk of screen-detected breast cancer. Meanwhile, age at menopause and nulliparity entailed a greater risk of tumors containing positive hormone receptors. Moreover, we found that screen-detected breast cancers were less likely to be TN tumors. In addition, BMI was related to an increased risk of breast cancer, regardless of the detection method and tumor phenotype.

There is little evidence available on the association of epidemiological risk factors with breast cancer according to method of detection. Unlike the present study, the study conducted by Sprague et al*.* [25]does not consider tumor phenotype in the analysis. This study reveals that the association of parity/nulliparity with screen-detected and non-screen-detected tumors varies, in line with our results. Nonetheless, they found no differences in the association with age at menopause between screen-detected and non-screen-detected breast cancers.

Previous analyses [29] found that the association of BMI and family history of breast cancer varied between interval BC and screen-detected BC. Although our study does not specifically look at interval breast cancers, we can assume that they are included in non-screen-detected BC, as defined in this paper. Our results show no variation in the association of family history with SD and NSD breast cancers, with no significant risk association in either case. Meanwhile, BMI is a known risk factor for cancer in general, and particularly for breast cancer in postmenopausal women [30], possibly due to increased levels of circulating endogenous estrogen [31]. Accordingly, our analysis reveals a risk association, though slight, between breast cancer and BMI. However, we found no variation in the association of BMI with SD and NSD breast cancers. A prior study comparing SD and interval BC shows that overweight women are more likely to have SD breast cancer [29], although weight-related barriers to mammography screening have been described previously [32]. Further analysis is needed to ascertain the associations between screen-detected and non-screen-detected breast cancers and BMI as well as family history of breast cancer.

Previous evidence has demonstrated a risk association between tumors expressing hormone receptors and reproductive factors [3–5, 33]. In the present study, similar results were observed for nulliparity (in postmenopausal women) and age at menopause, although we found no further associations with other reproductive variables that are also known risk factors for hormone-dependent tumors.

Considering the results according to detection method and phenotype, similarities can be observed in the association between screen-detected breast cancers and HR+ tumors with reproductive factors, specifically for nulliparity and age at menopause. This fact suggests that variation in breast cancer-risk factor associations by method of detection (screen-detected vs. non-screen-detected) could be explained by underlying prevailing phenotypes. Thus, the risk association of screen-detected tumors with these reproductive factors could be explained by the fact that they are predominantly hormone-sensitive tumors, which are in turn related to reproductive factors that modify endogenous hormone exposure [21, 34–36]. In line with these observations, several studies [23, 24, 37, 38] contribute further evidence that TN tumors are less frequent in breast cancers detected by screening mammography. Due to their rapid progression and shorter asymptomatic phase, these tumors often appear in the time lapse between two screening mammograms (2 years). In this regard, prevalence bias may be a limitation in this study, since HR+ tumors grow slowly and are thus more likely to be detected by screening than other tumor phenotypes.

Our results do not conclusively confirm associations with other known lifestyle-related risk factors [3, 4], such as tobacco use [39–43] and alcohol consumption [6, 44–47]; this could be due to the limitations of the study, which are outlined later in this section.

With regard to self-referred data (e.g., detection method), there may be a recall bias. Nonetheless, the impact was lessened thanks to the intervention of professional interviewers and the thorough design of the epidemiological questionnaire. Another limitation in this analysis may be the limited number of cases including information on phenotype, which makes stratification difficult.

The strengths of our study include its population-based design and the fact that it includes both opportunistic and organized screening, unlike most studies, which refer exclusively to the latter. As far as we know, this is one of the few studies conducted in a European context that analyzes breast cancer-risk factors according to detection method and tumor phenotype. Taking into account these results it would be advisable to consider detection method in future epidemiological studies on breast cancer. Moreover, further research on the differences between SD and NSD tumors, regarding prognostic and predictive tumor characteristics, could provide relevant information for patient management.

In conclusion, we found that screen-detected breast cancers are essentially associated with reproductive risk factors, and also with tumors including positive hormone receptors. Our results seem to indicate that the differences found in breast cancer-risk factor associations according to detection method can be attributed to predominating phenotypes, given that screening mammography mostly detects hormone-sensitive tumors which are susceptible to changes caused by reproductive factors.

## Supplementary Information

Below is the link to the electronic supplementary material.

Supplementary file1 (DOCX 17 KB)

## Data Availability

The database was registered with the Spanish data protection Agency, under Number 210267217118. Data are available under request.
