# Supplementary material for: Differences in breast cancer-risk factors between screen-detected and non-screen-detected cases (MCC-Spain study)
Source: Cancer Causes Control. 2021 Nov 24;33(1):125–36. doi: 10.1007/s10552-021-01511-4 (PMC8739309; doi:10.1007/s10552-021-01511-4)
Supplement: Supplementary file 1 — Supplementary file1 (DOCX 17 KB) [file 10552_2021_1511_MOESM1_ESM.docx]

Supplementary table

**Breast cancer-risk factors for overall sample, postmenopausal women, and parous women.**

|  | Cases vs. Controls | | |
| --- | --- | --- | --- |
|  | Overall sample | Postmenopausal | Parous women |
|  | OR^1^ (95%CI) | OR^2^ (95%CI) | OR^1^ (95%CI) |
| Tobacco use (ref: never) |  |  |  |
| Ever | 0.90 (0.72-1.12) | 0.91 (0.71-1.17) | 0.88 (0.69-1.12) |
| Alcohol consumption (g/day) | 1.01 (1.00-1.02) | 1.01 (1.00-1.02) | 1.01 (1.00-1.01) |
| BMI | 1.04 (1.02-1.07) * | 1.05 (1.02-1.08) * | 1.04 (1.01-1.07) * |
| Physical Activity (ref: active) |  |  |  |
| Inactive | 1.10 (0.88-1.37) | 1.11 (0.86-1.43) | 1.13 (0.89-1.44) |
| Fruits and vegetables intake (g/day) | 1.00 (0.99-1.00) | 1.00 (0.99-1.00) | 1.00 (1.00-1.00) |
| Age at menarche (ref:>12 years) |  |  |  |
| ≤ 12 years | 1.07 (0.87-1.32) | 1.07 (0.85-1.36) | 1.07 (0.85-1.34) |
| Nulliparity (ref: No) |  |  |  |
| Yes | 1.25 (0.94-1.65) | 1.54 (1.11-2.15) * |  |
| Age at menopause (ref: ≤50 years) |  |  |  |
| >50 years |  | 1.48 (1.16-1.89) * |  |
| Age at first birth |  |  | 1.02 (0.99-1.05) |
| Family history of BC (ref: no) |  |  |  |
| Yes | 1.32 (0.95-1.82) | 1.16 (0.80-1.68) | 1.36 (0.96-1.92) |

^1^OR adjusted for region, educational level, and menopausal status; ^2^OR adjusted for region; *p-value< 0.05
